# Prognostic value of carboxyhemoglobin in pediatric intensive care unit

**DOI:** 10.12669/pjms.38.7.5753

**Published:** 2022

**Authors:** Mehmet Alakaya, Ali Ertug Arslankoylu

**Affiliations:** 1 Mehmet Alakaya, Assistant Professor of Pediatric Intensive Care, Mersin University, Faculty of Medicine, Department of Pediatric Intensive Care Unit, Mersin, Turkey; 2 Ali Ertug Arslankoylu, Professor of Pediatric Intensive Care, Mersin University, Faculty of Medicine, Department of Pediatric Intensive Care Unit, Mersin, Turkey

**Keywords:** Carboxyhemoglobin, Pediatric Intensive Care Unit, Mortality

## Abstract

**Objectives:**

To determine the carboxyhemoglobin (COHb) levels of the patients admitted to the pediatric intensive care unit and investigate its relationship with prognosis.

**Methods:**

This retrospective observational study included patients aged one month to 18 years admitted to Mersin University Hospital pediatric intensive care unit from January 2020 to January 2021. Demographic characteristics, hospitalization causes, PRISM III, PELOD scores, hospitalization length, mechanical ventilation supports, transfusion needs, lactate and, SpCO levels of all patients were determined. SpCO levels of the excitus and surviving patients were compared, and the relationship with mortality was investigated.

**Results:**

Total 365 patients were included in the study. The median carboxyhemoglobin level of the excitus patients was statistically significantly higher when compared to the level of the surviving patients [(1.8(1.4-2,4) vs 0.65(0-1) p<0.001]). For mortality prediction, the cut-off point for SpCO, which was determined with 100% sensitivity and 96.5% specificity, was calculated as 1.3.

**Conclusion:**

Since SpCO levels are increased in critically ill children and correlate with increased PICU mortality, SpCO may be a predictive marker for prognosis in PICU.

## INTRODUCTION

Carboxyhemoglobin (COHb) is an indicator of the endogenous production of carbon monoxide (CO) by the Heme Oxygenase (HO)-1. Approximately 85% of endogenous CO is produced during the metabolism of heme by HO-1.^1^ HO-1 is the only inducible isoform among the two isoforms of HO.^2^ It is induced by hypoxia, oxidative stress, heavy metals, and also by cytokines.^2^ CO competitively binds to oxygen binding sites on haemoglobin and impedes oxygen transport in the blood. HO-1 activity cleaves heme to form CO, biliverdin and iron, which play a concerted action in cytoprotection against oxidative stress and in the modulation of cell proliferation and differentiation.^3^

Fazekas et al. demonstrated the association between low COHb and mortality in adult intensive care unit (ICU) patients.^4^ We hypothesise that similar responses as seen in adult patients should be expected in patients admitted to the pediatric intensive care unit (PICU). Moreover, increased HO-1 activity in children has been described in disease states such as obesity^5^ and haemolysis.^6^ But as far as we know, there are limited studies that have investigated the association between COHb levels and PICU outcomes in the literature.^7^ That’s why in this study, we aimed to investigate the COHb levels in children admitted to PICU and to show the association between COHb and PICU outcomes. Pulse CO-oximetry is a continuous and non-invasive method of measuring the levels of various blood constituents, including carbon monoxide (SpCO). Despite the percentage of blood carboxyhemoglobin is frequently shown with CO oximetry in blood gas analysis, SpCO has been measured using pulse CO-oximetry in late years and has been shown that SpCO levels are correlated with COHb levels measured in blood gas devices.^8^,^9^ COHb levels were measured as SpCO using the pulse co-oximeter method in this study.

## METHODS

In this retrospective observational study, children aged 1 month-18 year admitted to the 12-bed pediatric intensive care unit between January 1st, 2019, and January 1st, 2020, were included in this retrospective observational study. Patients with hemolysis and CO poisoning were excluded. Approval was obtained from Mersin University Clinical Research Ethics Committee. (Approval No: 2021/601).

Demographic characteristics, hospitalization cause, PRISM III, PELOD scores, hospitalization length, mechanical ventilation support, transfusion requirements, and blood gas lactate levels at hospitalization (0. hour) and SpCO levels at hospitalization (0. hour) of all patients admitted to the PICU were obtained from the hospital information management system (NUCLEUS v9.35.20). The accuracy of the information acquired was confirmed by comparing it with the patient files. Mortality rates and hospitalization duration in the PICU were calculated as prognostic indicators of the patients. In order to predict disease severity and seriousness, PELOD and PRISM III scoring systems were used. After all the data was collected, the patients were divided into two as survivors and exitus according to prognosis, and SpCO levels and other parameters were compared.

As soon as patients were admitted to the PICU, SpCO levels were measured by a physician trained in the use of the Massimo Root (Radical-7 Rainbow SET Pulse CO-Oximeter; Masimo Corporation; Irvine, CA) device. The appropriate sized finger sensor was placed on the patient’s 3rd or 4th fingers during the measurement in accordance with the manufacturer’s recommendations and connected to the pulse co-oximeter. Pre-measurement calibration and measurement techniques were performed in line with the device usage instructions.

For lactate measurements, blood gas measurements were carried out using capillary blood. Blood gas measurements were carried out with the ABL800 FLEX (RADIOMETER, 2019, Denmark) device.

For statistical analysis of the data, SPSS 23.0 package program was used. Categorical measurements were summarised as numbers and percentages, and continuous measurements as mean deviation, and minimum-maximum. The conformity of the variables to the normal distribution was examined using the Kolmogorov-Smirnov/Shapiro-Wilk Tests. To compare categorical variables, the Chi-square test and Fischer’s Precision Test were used. Mann Whitney U test was used for binary variables in groups that did not fit the normal distribution. Sensitivity and specificity were evaluated based on the SpCO values of the patients, and the cut-off value was determined by examining the area under the receiver operating characteristic (ROC) in the study. Statistical significance level was taken as 0.05 in all tests.

## RESULTS

During the study period, a total of 371 patients were hospitalized in the PICU. Six of these patients were excluded from the study due to hemolysis and CO poisoning. A total of 365 patients were included in the study. The characteristic features of the patients are given in Table-I. The majority (41.4%) of the patients included consisted of patients with respiratory and circulatory failure. The mean SpCO of the patients was found to be 0.82±0.51.

**Table I:** Characteristics of the patients included in the study.

		Frequency (n)	Percentage (%)
Gender	Female	183	50,1
	Male	182	49,9
Causes of Hospitalization	Respiratory and circulatory failure	151	41,4
	Neurological diseases	64	17,5
	Trauma	88	24,1
	Hematological and oncological diseases	37	10,1
	Kidney failure and nephrological diseases	14	3,8
	Liver failure and gastrointestinal diseases	11	3,0
Surgery	No	304	83,3
	Yes	61	16,7
Underlying Disease	No	163	44,7
	Yes	202	55,3
Mechanical Ventilation	No	303	83,0
	Yes	62	17,0
Transfusion	No	329	90,1
	Yes	36	9,9
Result	Survived	318	87,1
	Exitus	47	12,9

		*Mean±SD*	*Med (Min-Max)*

Age (months)		77,01±71,0	48 (2-212)
PRISM III		7±10,21	4 (0-60)
PELOD		6,73±8,8	4 (0-42)
SpCO		0,82±0,51	0,8 (0-2,4)
Lactate (mmol / L)		2,68±1,79	2.3 (0.1-16.1)
Length of stay in PICU (days)		8,43±12	4 (1-85)

PRISM; Pediatric risk of mortality score, PELOD; Pediatric logistic organ dysfunction score, SpCO; Carboxyhemoglobin.

In case the SpCO values were compared in line with the age groups of the patients and the PICU admission causes, no difference was found in the SpCO values according to the age and the hospitalization cause. (Table-II).

**Table II:** Comparison of SpCO levels according to age and causes of hospitalization.

	SpCO		p

	Mean±SD	Med (Min-Max)	
***Age group***			
< 12 months	0,85±0,51	0.9 (0-1.9)	0.882
Between 12-36 months	0,81±0,50	0.8 (0.1-2)	
> 36 months	0,82±0,52	0.7 (0-2.4)	
***Cause of hospitalization***			
Respiratory and circulatory failure	0,82±0,55	0.7 (0-2.4)	0.618
Neurological diseases	0,73±0,44	0.7 (0-2)	
Trauma	0,86±0,52	0.7 (0-2.1)	
Hematological and oncological diseases	0,87±0,50	0.9 (0.1-2.2)	
Kidney failure and nephrological diseases	0,77±0,46	0.75 (0.1-1.9)	
Liver failure and gastrointestinal diseases	0,95±0,43	0.9 (0.3-1.8)	

Characteristics and SpCO levels of the patients included in the study were compared by dividing them into two groups as survivors and those who were excitus. There was no significant difference in terms of gender (p=0.283), hospitalization cause (p=0.331), underlying disease (p=0.532) and age (p=0.380), and outcome findings (p>0.05) between the two groups. While the incidence of surgical presence (p=0.005) was found to be significantly lower in patients who died (p<0.05), the incidence of mechanical ventilation (p<0.001) and transfusion (p<0.001) was found to be significantly higher when compared to those in the transferred/discharged group (p<0.05). Findings of PRISM III (p=<0.001), PELOD (p=<0.001), SpCO (p=<0.001), Lactate (p=<0.001), and ICU stay (p=0.032) of excitus patients were determined to be significantly higher than the patients in the transferred/discharged group (p<0.05). The median SpCO level of the exitus patients was statistically significantly higher when compared to the level of the surviving patients [(1.8(1.4-2,4) vs 0.65(0-1) p<0.001]). (Table-III)

**Table III:** Comparison of the characteristics of surviving and exitus patients.

		R		p

		Survive	Exitus	

		n(%)	n(%)	
Gender	Female	156(49,1)	27(57,4)	0.283
	Male	162(50,9)	20(42,6)	
Cause of hospitalization	Respiratory and circulatory failure	127(39,9)	24(51,1)	0.331
	Neurological diseases	60(18,9)	4(8,5)	
	Trauma	74(23,3)	14(29,8)	
	Haematological and oncological diseases	34(10,7)	3(6,4)	
	Kidney failure and nephrological diseases	13(4,1)	1(2,1)	
	Liver failure and gastrointestinal diseases	10(3,1)	1(2,1)	
Surgery	No	259(81,4)	45(95,7)	0.014
	Yes	59(18,6)	2(4,3)	
Underlying disease	No	144(45,3)	19(40,4)	0.532
	Yes	174(54,7)	28(59,6)	
Mechanical vent	No	286(89,9)	17(36,2)	<0.001
	Yes	32(10,1)	30(63,8)	
Transfusion	No	303(95,3)	26(55,3)	<0.001
	Yes	15(4,7)	21(44,7)	

		Survive	Exitus	p

		Med (Min-Max)	Med (Min-Max)	

Age (months)		48 (2-212)	72 (2-209)	0.380
PRISM III		4 (0-36)	24 (2-60)	<0.001
PELOD		3 (0-32)	24 (2-42)	<0.001
SpCO		0,65 (0-1)	1,8 (1,4-2,4)	<0.001
Lactate (mmol / L)		2,2 (0,1-4,8)	5,3 (2,1-16,1)	<0.001
Length of stay in PICU (days)		4 (1-78)	7 (1-85)	0.032

*p<0.05, ROC Curve, (AUC: Area Under the Curve, Se: Sensitivity, Sp: Specificity, PPV: Positive Comparison value, NPV: Negative Comparison Value).

**Fig.1:**
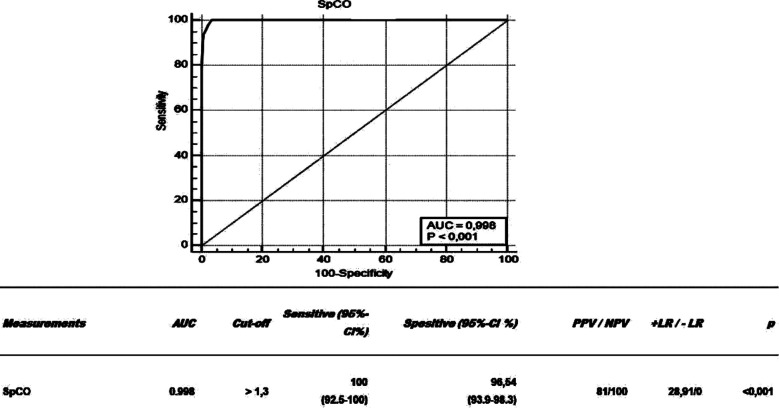
Representation of SpCO and exitus relationship with ROC curve and examination of the findings of the ROC analysis of the patients who died in terms of SpCO.

To establish a cut-off for SpCO, ROC analysis and ROC curve were created with the values of the patients who exitus. (Fig.1) As a result of the analysis, the area under the ROC curve was evaluated as 99.8%. In other words, it was understood that the cut-off value obtained gave the correct answer at the rate of 99.8%. In line with the cut-off value obtained, if the SpCO is above 1.3, it is predicted that the patient has a mortality risk with 100% sensitivity and 96.54% specificity. (Fig.1)

## DISCUSSIONS

The optimal COHb value in patients hospitalised in the PICU is unknown. Even though high COHb levels are fatal in CO poisonings, it has been reported that the range of 0.6%-1.8% may be optimal in critically ill patients hospitalized in adult ICU.^1^ The mean SpCO value of PICU patients at admission was 0.82% in our study. This value was lower than the value (1.00%) reported by Chavla et al. in their study on children.^7^ Hovewer all the patients in Chavla et al’s study were more severe patients than ours, receiving mechanical ventilation support. That’s why we thought that their COHb levels were higher when compared to those in our study. At the same time we think that using the different COHb measurement methods in these studies may be the cause of this difference in COHb levels. On the other hand, the mean COHb level that was reported by Fazekas et al. in a study on adult intensive care patients, was higher than COHb value in our study.^4^ We think that while comparing COHb values, it should not be ignored that smoking may have an increasing effect on COHb levels in adult patients.

The SpCO levels of the patients hospitalized in the PICU were determined to be similar according to the causes of hospitalization in our study. On the other hand, Fazekas et al. reported, in their study with adult ICU patients, that COHb levels were highest in patients with renal and gastrointestinal disease and lowest in patients with neurological disease.^4^ It is thought that this difference may be due to the fact that the indications for ICU hospitalization of adult and pediatric patients are not similar. Nevertheless, to obtain pathophysiological conclusions, there is a need for studies with larger populations comparing the COHb levels of patients hospitalized in the PICU for different indications.

In this study, in the case of comparing SpCO levels according to age groups, it was observed that there was no significant difference between the groups. In the study of Chawla et al. conducted on PICU patients, it was reported that young children, especially infants, have higher COHb values.^7^ The explanation of this may be the fact that infants had higher PIM scores in the study of Chawla et al., and therefore they were more severe patients. Even though SpCO values under one year of age were found to be higher than other age groups, the difference was not statistically significant in our study.

Increased expression of HO-1 has been demonstrated in critically ill patients, including those with SIRS and acute respiratory distress syndrome previously.^10^,^11^ It has been hypothesised that HO-1 induction may be beneficial since products of its catabolic activity, including carbon monoxide (CO), biliverdin, and bilirubin, possess anti-inflammatory and antioxidant properties.^12^,^13^ By contrast, some studies have demonstrated that excessive HO-1 activity is deleterious, possibly through the liberation of molecular iron, the final prooxidant product of heme degradation.^14^ Since HO-1 is highly induced by oxidative stress, and it may be a biomarker for oxidative stress and inflammatory response. Furthermore, arterial COHb levels are elevated in patients following cardiac surgery,^15^ in those with inflammatory lung conditions,^12^ and in the critically ill.^16^

The fact that the median SpCO levels of the exitus patients were statistically significantly higher than the surviving patients was supporting the hypothesis of our study. The high SpCO levels of the patients at the time of admission suggests that there is increased oxidative stress and inflammation from the beginning in the patients who died. Actually Hara et al reported the correlation between the increased carboxyhemoglobin levels and pulmonary inflammation which was consistent with our results.^17^ Even though Chawla A et al. was not able to find a relationship between COHb level at first admission and survival, they reported that patients who exitus had higher COHb levels during follow-up.^7^ As only one measurement was made in our study, we cannot report a conclusion about the course of SpCO levels, but we estimate that SpCO levels may have increased with increased oxidative stress in exitus patients. To clarify this issue, it would be useful to conduct larger studies with serial measurements. In the study of Chavla et al., it was reported that a high COHb level was, in addition to mortality, associated with a longer mechanical ventilation duration.^7^ Similarly, in our study, patients with high COHb levels had higher rates of mechanical ventilation. This supports the hypothesis that patients with more severe general condition have higher COHb levels. On the other hand, the correlation between PRISM III, PELOD scores, and lactate and SpCO level of exitus and surviving patients at the time of admission suggest that SpCO may be an objective parameter that can be used to predict prognosis in critically ill patients. Similar to our study in recent studies carboxyhemoglobin level was reported as a useful biomarker to predict prognosis in COVID-19 patients^18^ and burn patients.^19^

Our study is original as it is the first study in the literature to show the relationship between COHb level and mortality in patients hospitalized in the PICU using the pulse CO-oximetry method. Since it is a non-invasive and easy method to measure the COHb level with the pulse CO-oximetry method, we think that the use of COHb can be a useful parameter to predict the prognosis in PICU.

### Limitations

SpCO levels were measured only at the time of admission in our study. Since we did not make serial measurements it was not possible to examine whether there was a difference between the course of SpCO levels of the patients who were exitus and those who survived. Another limitation of this study is not to take account the haemoglobin levels, which were reported to affect COHb levels in previous studies.^20^,^21^

## CONCLUSION

SpCO levels at the time of admission of the patients who died in the PICU were significantly higher than those of the surviving patients. SpCO levels at the time of admission to the PICU may be one of the useful parameters to predict mortality in critically ill children who were admitted to PICU.

### Authors’ Contribution:

**MA:** Conceived, designed, Did statistical analysis & editing of manuscript, Manuscript writing, Final approval of manuscript. He is also responsible for the integrity and accuracy of the study.

**AEA:** Did data collection, manuscript writing.
